# Nonlinear statistical damage constitutive model of granite based on the energy dissipation ratio

**DOI:** 10.1038/s41598-022-09503-3

**Published:** 2022-03-31

**Authors:** Xianliang Wang, Jianhai Zhang, Li Qian, Tianzhi Yao, Zuguo Mo, Jianhua He, Ru Zhang

**Affiliations:** 1 State Key Laboratory of Hydraulics and Mountain River Engineering, College of Water Resources and Hydropower, Sichuan University, Chengdu, 610065 China; 2 Sichuan Metallurgical Geological Survey and Design Group Corporation Limited, Chengdu, 610051 China; 3 Power China Chengdu Engineering Corporation Limited, Chengdu, 610072 China

**Keywords:** Civil engineering, Geology

## Abstract

The stress–strain curves and mechanical properties of Shuangjiangkou granite were obtained using five groups of conventional triaxial tests under various confining pressures using MTS815 rock test equipment. From the microscale, mesoscale, and macroscale perspectives, four types of mechanisms that contribute to energy dissipation during granite deformation were investigated. Based on the energy dissipation ratio, a new approach for estimating crack closure stress and damage stress is proposed. The energy dissipation ratio was substituted into the Weibull distribution function, and then a new nonlinear statistical damage constitutive model of granite based on the energy dissipation ratio was constructed after Biot’s theory was modified per the Lemaitre strain equivalence principle. By comparing experimental data with theoretical values estimated by the model, the model’s rationality and correctness were confirmed.

## Introduction

The mechanical behaviour and characteristics of rock under diverse external loads have always been a subject of interest to geotechnical engineers^1–7^. The damage, deformation, failure and post-failure deformation of rock can be directly used for large rock engineering design. The description of rock strength and deformation behaviour (especially the study of constitutive relationships) is the basis for evaluating the stability of rock engineering structures^8–10^. In general, the most effective technique to investigate the rock deformation process and failure mechanism is to determine the complete stress–strain relationship. According to a significant number of studies, the complete stress–strain curve of brittle rock can be split into five stages, namely, compression closure of primary cracks, linear elastic deformation, stable crack growth, unstable crack growth, and post-peak failure deformation, by four stress thresholds^11–13^. It is critical to determine stress thresholds at each step of rock development to investigate its strength and deformation^14,15^. To date, there have been several approaches for determining stress thresholds, but none for determining the thresholds in terms of the energy evolution law.

The deformation and fracture of rocks is the outcome of an irreversible dissipation process in which energy is converted from one form to another and entropy is generated, according to thermodynamics^16^. Detailed investigation of energy transmission and transformation during the evolution of rock damage can more accurately reflect the law of rock damage. However, in many studies^17–20^, the mechanism of energy dissipation at the trans-scale microscopic, mesoscopic, and macroscopic levels of damage and failure progression in brittle rock has not been investigated in great detail. An acoustic emission (AE) is defined as a transient elastic wave generated by the rapid release of energy within a material, therefore, it can be used to predict rock failure as well as to study earthquake processes^21–28^. Brittle rock materials are naturally heterogeneous, with macroscopic discontinuous structures (such as flaws, joints, cracks, and weak surfaces) as well as internal composite microscopic structures (such as different mineral compositions and randomly distributed particles). Even rocks that appear intact contain primitive microcracks and microvoids. Under the action of external factors (including force, temperature, electricity, magnetism, radiation, etc.), a large number of new microfissures and micropores will form inside the rock, and these microfissures will nucleate, cross and merge, resulting in gradual damage and even destruction of the rock^29,30^. In damage mechanics^31–35^, all microdefects are continuous, and their effect on the material is expressed by one or more internal field variables (i.e., damage variable), but these microdefects are discrete and random in nature.

As a result, the constitutive relationship of rock may be investigated from a statistical perspective, and the constitutive theory and evolution equation of materials with damage can be studied using the random distribution function.

The Weibull distribution is one of the most extensively used statistical models for heterogeneous brittle materials, and the statistical uniformity evaluation approach paired with the Weibull distribution has been widely utilized to analyse brittle material failure^36–39^. The majority of current studies use the mechanical or deformation properties of rock as the foundation for developing a statistical damage constitutive equation. Krajcinovic et al.^32^ used the Weibull distribution function to establish the damage equation for brittle materials and examined the related parameters. Based on the statistical evolution equation, Cao et al.^40^ developed a statistical damage constitutive model for rock strain softening and hardening under conventional triaxial compression. Pourhosseini et al.^41^ suggested a constitutive model based on the Mohr–Coulomb failure criterion that may account for both rock strain-softening and dilation behaviour. Molladavoodi et al.^42^ assumed that the shear and bulk modulus of rock elements follow the Weibull distribution and then used a constitutive model to link the Mohr–Coulomb strain-softening model to a commercial discrete element method (DEM) code. Although these studies used mathematical statistics to develop rock constitutive models, none of them are based on energy evolution rules, which could better describe the nature of rock deformation and fracture. Despite the fact that the rule of energy evolution can in essence describe the process of rock deformation and failure, few studies have included it in the damage constitutive relationship of mesoscopic statistics.

The stress–strain relationship curves for five groups of rock samples were acquired using MTS815 rock triaxial test equipment. This equipment was used to conduct conventional triaxial compression tests of granite under various confining pressures to investigate the relationship between energy dissipation and the damage constitutive equation of Shuangjiangkou granite under different in situ stresses. From the microscale, mesoscale, and macroscale perspectives, four types of energy dissipation mechanisms during rock damage evolution were analysed. A new approach for calculating stress thresholds (crack closure stress and damage stress) based on the energy dissipation ratio is provided. The energy dissipation ratio was substituted into the Weibull distribution function, and generalized Hooke’s law was modified per the Lemaitre strain equivalence principle^31^, resulting in a new nonlinear statistical damage constitutive model for granite. The model’s rationality and correctness were verified by comparing the theoretical values derived by the model with the experimental data.

## Test method

### Test samples

**Figure 1:**
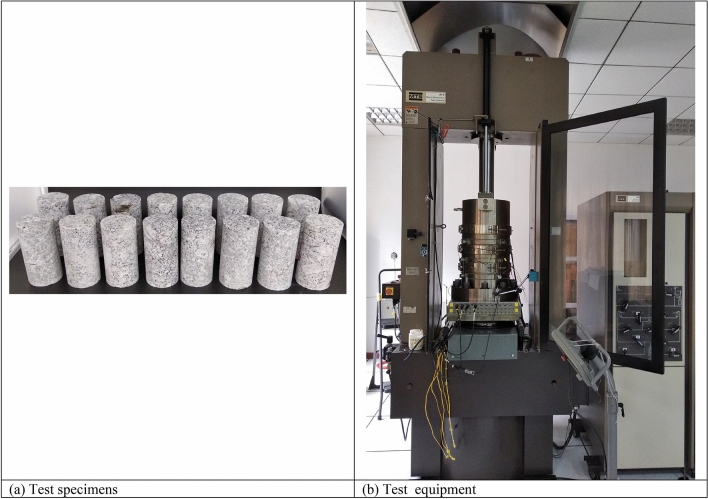
Test samples and test equipment.

The rock samples utilized in this test came from the guide tunnel of Shuangjiangkou Hydropower Station’s main and auxiliary workshops in China. The tunnel was dry and the value of the in situ pore pressure was 0, so pore pressure can be neglected. It took 15 days from the tunnel excavation to the extracted samples to be sent to the laboratory. After the irregular samples were sent to the laboratory, the samples were processed into cylindrical specimens with a diameter of 50 mm and a height of 100 mm in accordance with the method suggested by the International Society for Rock Mechanics^43^, as shown in Fig. 1a and Table 1. The specimens were dried in a constant temperature ventilated drying oven at 105 °C for 24 h and subsequently cooled in a sealed desiccator, followed by a series of tests as described below.

**Table 1:** Shape and mechanical parameters of granite samples.

*σ*_3_ (MPa)	Density (g/cm^3^)	Height (mm)	Diameter (mm)	*ν*	*E* (GPa)
1	2.72	99.13	50.75	0.08	53.02
5	2.70	100.22	50.26	0.10	54.30
10	2.73	99.93	50.86	0.17	56.29
30	2.72	99.22	50.85	0.18	63.67
40	2.71	99.26	50.01	0.31	64.30

### Test equipment

The tests were conducted using the rock mechanics test equipment (MTS815 Flex Test GT) of Sichuan University’s College of Water Resources and Hydropower Engineering, as illustrated in Fig. 1b. The major technical parameters of the test equipment are as follows: the maximum axial force is 4600 kN; the maximum confining pressure is 140 MPa; the operating temperature is 20–200 °C; the axial and circumferential extensometer resolutions are ± 4 mm and − 2.5 to 8 mm, respectively; and the measurement accuracy is 0.5%.

### Test scheme

This study included five groups of conventional tests of granite, with designed confining pressures of 1, 5, 10, 30, and 40 MPa; these pressures were chosen based on the stress state of the rock mass at different depths from the underground powerhouse to the cave wall in Shuangjiangkou.

The experimental procedure during each test was as follows^43^: (1) an external load was supplied to the predetermined hydrostatic pressure at a rate of 0.05 MPa/s and allowed to stabilize; (2) an axial load was applied at a rate of 0.5 MPa/s while maintaining a constant confining pressure until the rock sample failed at the end of the test; and (3) the maximum axial force on the sample was measured, as well as the corresponding confining pressure.

## Mechanical characteristics

### Total stress–strain curve

The volumetric strain *ε*_*v*_ of a cylindrical sample exposed to axial loading with a confining pressure can be calculated with the recorded axial strain *ε*_1_ and lateral strain *ε*_3_.

**Figure 2:**
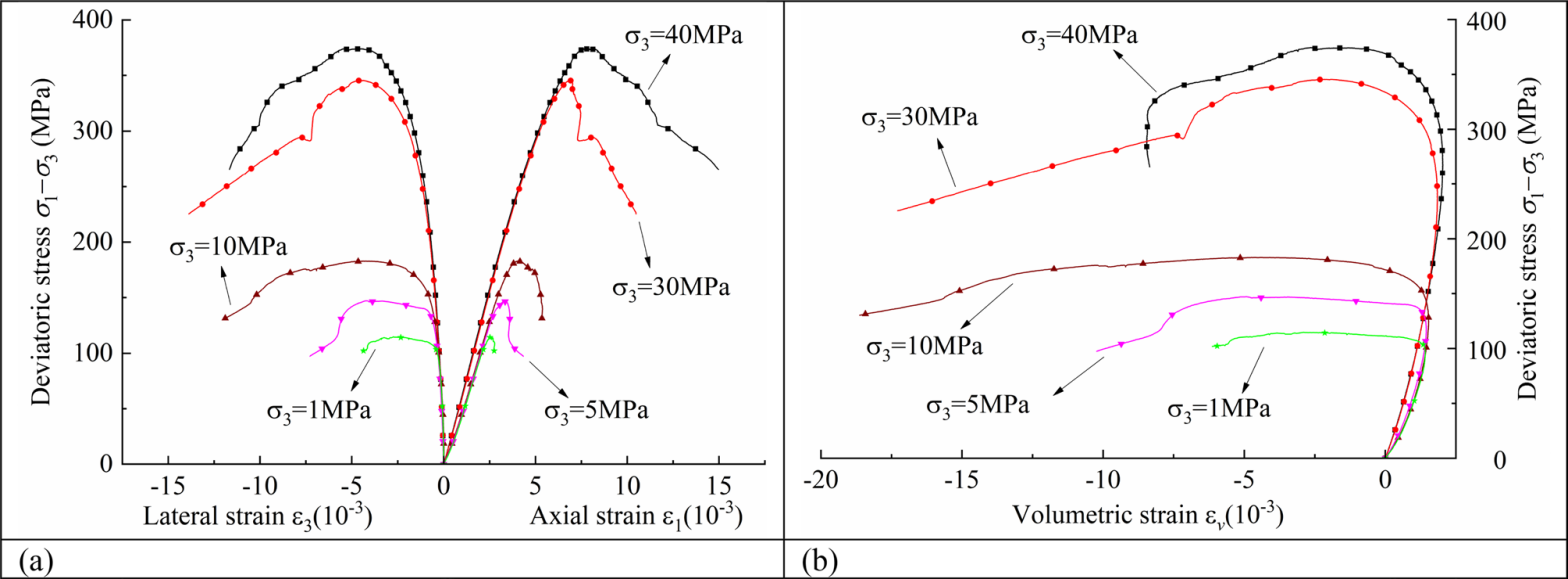
The conventional triaxial deviatoric stress–strain curves under various confining pressures.

The axial, lateral, and calculated volumetric strains vs. the applied deviatoric stress (*σ*_1_–*σ*_3_) can be plotted to follow the route of the failure of a rock sample. Figure 2 shows five groups of conventional triaxial compression test curves for granite with various confining pressures. The increase in confining pressure has the following effects on rocks; that is, the peak deviatoric stress, which represents compressive strength, increases; the elastic limit increases; the deformation corresponding to the peak stress increases; and the rock properties also change, with plasticity and strain hardening becoming more apparent.

### Strength and confining pressure

**Figure 3:**
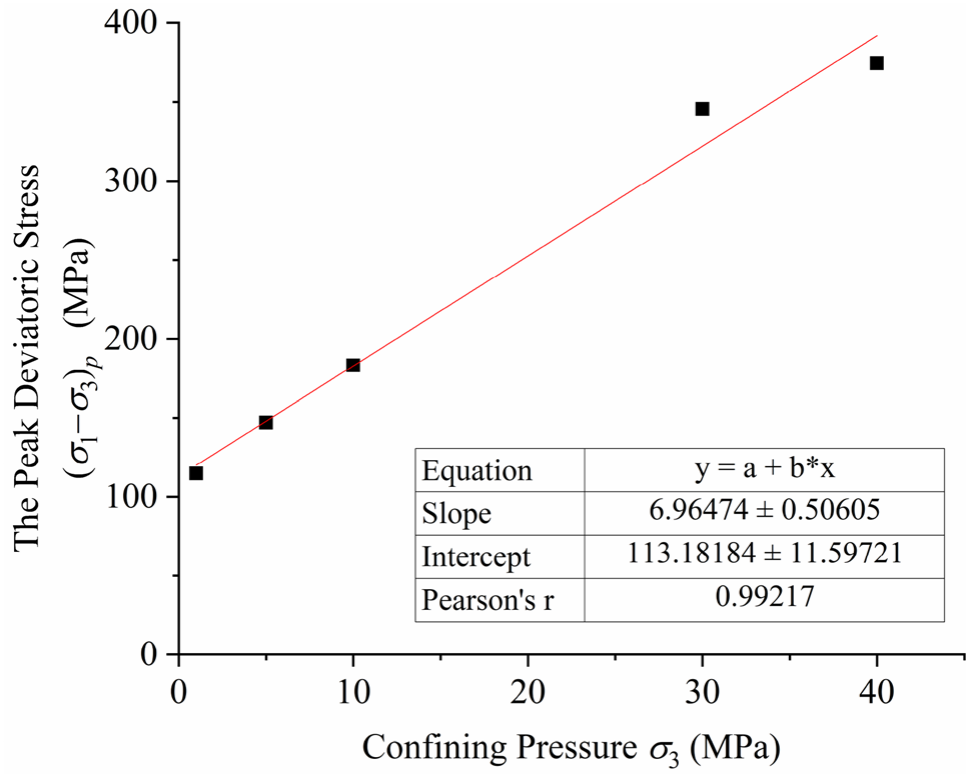
The relationship between the confining pressure (*σ*_3_) and the peak deviatoric stress (*σ*_1_–*σ*_3_)_*p*_*.*

Experiments have shown that confining pressure may greatly improve the strength of most rocks. The axial peak strength of rock continues to rise as the confining pressure increases. Equation (1) and Fig. 3 show the relationship between confining pressure (*σ*_3_) and peak deviatoric stress (*σ*_1_–*σ*_3_)_*p*_. There is a very obvious linear relationship between the two values, and the slope and intercept of the line are 6.96 and 113.18, respectively.

1 $$\left( {\sigma_{1} - \sigma_{3} } \right)_{p} = 113.18 + 6.96\sigma_{3} .$$

### Failure characteristic

**Figure 4:**
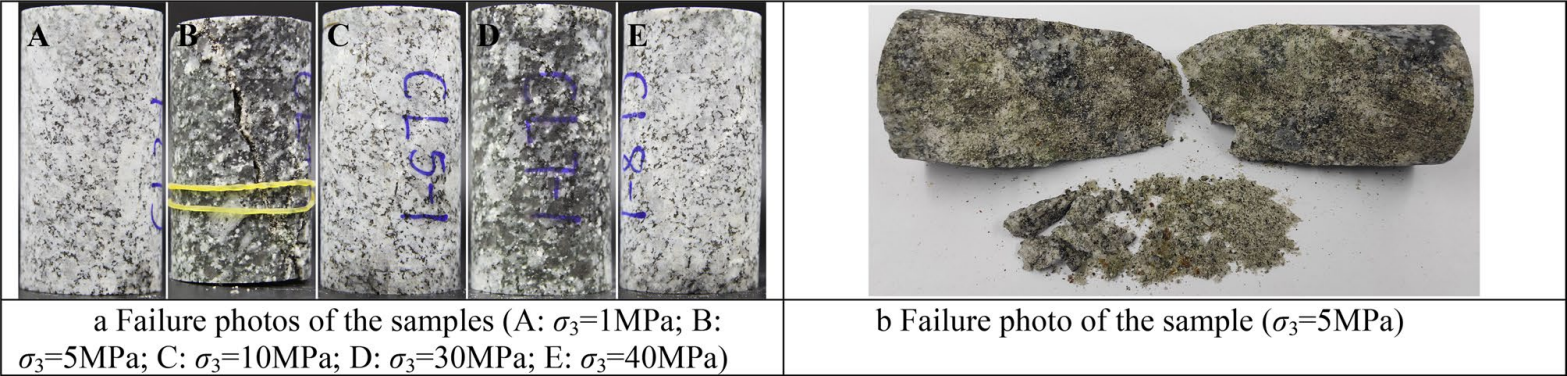
(**a**) Failure photos of the samples (A: *σ*_3_ = 1 MPa; B: *σ*_3_ = 5 MPa; C: *σ*_3_ = 10 MPa; D: *σ*_3_ = 30 MPa; E: *σ*_3_ = 40 MPa). (**b**) Failure photo of the sample (*σ*_3_ = 5 MPa).

The form of rock failure varies depending on the loading state. The failure of rocks is mainly in the form of tensile failure, splitting failure, shear failure and plastic flow. Figure 4 shows the failure photos from the conventional triaxial compression tests of Shuangjiangkou granite under various confining pressures. The failure of rock samples is mainly shear failure of oblique sections.

## Energy evolution and deformation characteristics

### Analysis of energy dissipation mechanism

As a geological structural material, rock is composed of one or more minerals. Due to the different physical properties, diagenetic conditions and processes of the compositions, the internal structure of rocks is very different, showing heterogeneity. There are numerous randomly distributed defects in rock materials at the microscale, mesoscale and macroscale levels, which cause the rocks to become damaged bodies. On the microscale, all deviations from the ideal lattice structure in the crystal configuration are called crystal defects. Dislocation is one of the most important kinds of defects whose slip movement causes crystal plastic deformation. The study of crystal plasticity, damage, strength, and other structural sensitivity problems relies heavily on dislocation theory^44,45^. On the mesoscale, the defects are mainly microvoids and microcracks whose nucleation and movement cause the damage evolution of materials. On the macroscale, the defect is a macroscopic crack and hole, which is common to our naked eye, and the appearance of macrodefects represents the beginning of material rupture. At a larger observation scale, defects are characterized by geological faults and large tectonic belts.

The energy absorbed by rock as a result of an external load can be split into two categories. Releasable elastic strain energy is held in one component, whereas dissipated energy is dissipated in the other. The irreversible alteration of the internal structure of rock materials is always followed by an energy dissipation process. The energy dissipation mechanism of rock materials is very complex and can be roughly divided into four types. Two main energy dissipation mechanisms are crystal plastic deformation and damage evolution. From the perspective of macroscopic mechanical behaviour, these mechanisms correspond to irreversible deformation and stiffness reduction; from the perspective of the microscopic structure of crystal materials, plastic deformation corresponds to crystal dislocation, and damage evolution corresponds to the nucleation and movement of microcracks^46,47^. Crystal plastic deformation and damage evolution are two types of closely related and interactive mechanisms. An inelastic deformation process generally has both types of mechanisms, and pure plasticity or pure damage is extremely rare. From a physical view, the two dissipative processes are essentially different; hence, plasticity and damage should be regarded as two independent energy dissipation mechanisms. During the loading process, rock materials contain a large number of microvoids and microcracks, the nucleation and movement of which are the dominant aspects of the energy dissipation mechanism, while crystal dislocation is a minor aspect. The friction of the fracture surface is also an important energy dissipation mechanism for rock materials, which is often the dominant energy dissipation mechanism under compression and shear conditions. Dilatancy caused by the roughness of the fracture surface is also an important aspect of energy dissipation, and the rock will show obvious dilatancy in the failure stage.

### Energy calculation principle

**Figure 5:**
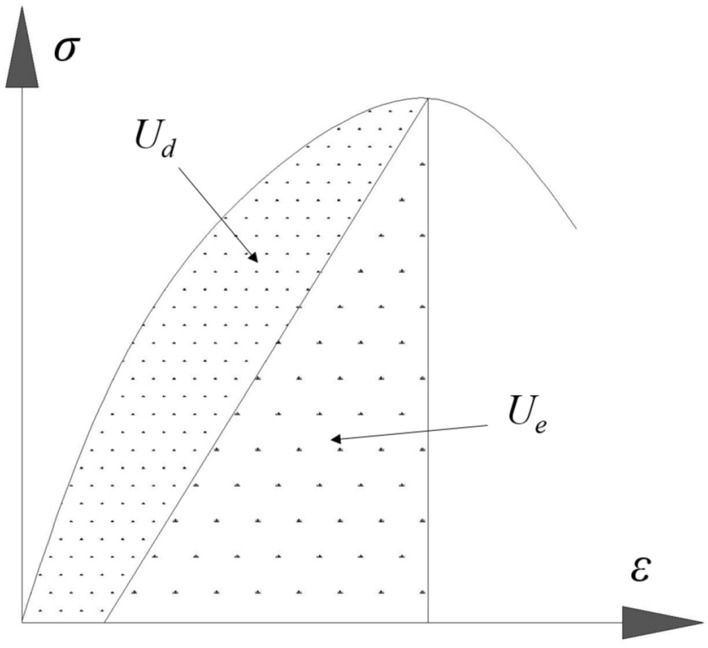
The relationship between dissipated energy and elastic strain energy.

If the conventional triaxial compression test system of rock is considered a closed system and the pressure system has no loss of external heat transfer during the process of loading rock samples, then the total energy generated by external work on rock samples can be calculated using Eq. (2)^16^.

2 $$U = U_{d} + U_{e} ,$$

where *U*, *U*_*d*_ and *U*_*e*_ represent the total absorbed energy density, dissipated energy density and released elastic strain energy density, respectively. The denser dotted area under the stress–strain curve reflects dissipated energy, while the sparser dotted area shows releasable elastic strain energy contained in rocks, as shown in Fig. 5.

Equation (3) gives the elastic strain energy density based on elastic theory.

3 $$U_{e} = \frac{1}{2}\left( {\sigma_{1} \varepsilon_{1}^{e} + 2\sigma_{3} \varepsilon_{3}^{e} } \right),$$

where *σ*_1_, *σ*_3_, *ε*_1_^*e*^ and *ε*_3_^*e*^ are the axial stress, lateral stress, elastic axial strain, and elastic lateral strain, respectively.

The elastic axial strain *ε*_1_^*e*^ and elastic lateral strain *ε*_3_^*e*^ can be obtained from Eqs. (4) and (5), respectively, based on Hooke’s law.

4 $$\varepsilon_{1}^{e} = \frac{1}{E}\left( {\sigma_{1} - 2\nu \sigma_{3} } \right),$$

5 $$\varepsilon_{3}^{e} = \frac{1}{E}\left[ {\sigma_{3} - \nu (\sigma_{1} + \sigma_{3} )} \right],$$

where *ν* and *E* are Poisson’s ratio and the elastic modulus, respectively, which can be calculated from the linear elastic section of the stress–strain curve, as shown in Table 1.

Equation (6) can be used to calculate the elastic strain energy density by substituting Eqs. (4) and (5) into Eq. (3).

6 $$U_{e} = \frac{1}{2E}\left[ {\left( {\sigma_{1} } \right)^{2} + 2\left( {1 - \nu } \right)\left( {\sigma_{3} } \right)^{2} - 4\nu \sigma_{1} \sigma_{3} } \right].$$

The total absorbed energy density in conventional triaxial tests can be calculated by Eq. (7)^48,49^.

7 $$U = U_{0} + U_{1} + U_{3} ,$$

where *U*_0_ is the energy density due to hydrostatic loading, *U*_1_ is the energy density due to axial compression by *σ*_1_ after hydrostatic pressure, and *U*_3_ is the energy density due to lateral compression by *σ*_3_ after hydrostatic pressure. *U*_0_, *U*_1_, and *U*_3_ can be determined by Eqs. (8)–(10)^48,49^.

8 $$U_{0} = \frac{3(1 - 2\nu )}{{2E}}\left( {\sigma_{3} } \right)^{2} ,$$

9 $$U_{1} = \int {\sigma_{1} d\varepsilon_{1} = \sum\limits_{i = 0}^{n} \frac{1}{2} } (\varepsilon_{1(i + 1)} - \varepsilon_{1i} )(\sigma_{1(i + 1)} + \sigma_{1i} ),$$

10 $$U_{3} = 2\int {\sigma_{3} d\varepsilon_{3} = \sum\limits_{i = 0}^{n} {(\varepsilon_{3(i + 1)} - \varepsilon_{3i} )(\sigma_{3(i + 1)} + \sigma_{3i} )} } ,$$

where *σ*_1*i*_, *ε*_1*i*_, *σ*_3*i*_, and *ε*_3*i*_ are the axial stress, axial strain, lateral stress, and lateral strain at point *i* on the stress–strain curve, respectively.

### Energy analysis

Under external loading, the native microvoids and microcracks randomly distributed in rock first close; then a stress concentration will be produced at the tip. When the stress concentration exceeds the critical value, the crack of microcracks will occur and expand. With increasing load, microcracks will bifurcate and converge, forming a macroscopic fracture surface and even instability failure. During the process, energy is constantly dissipated.

**Figure 6:**
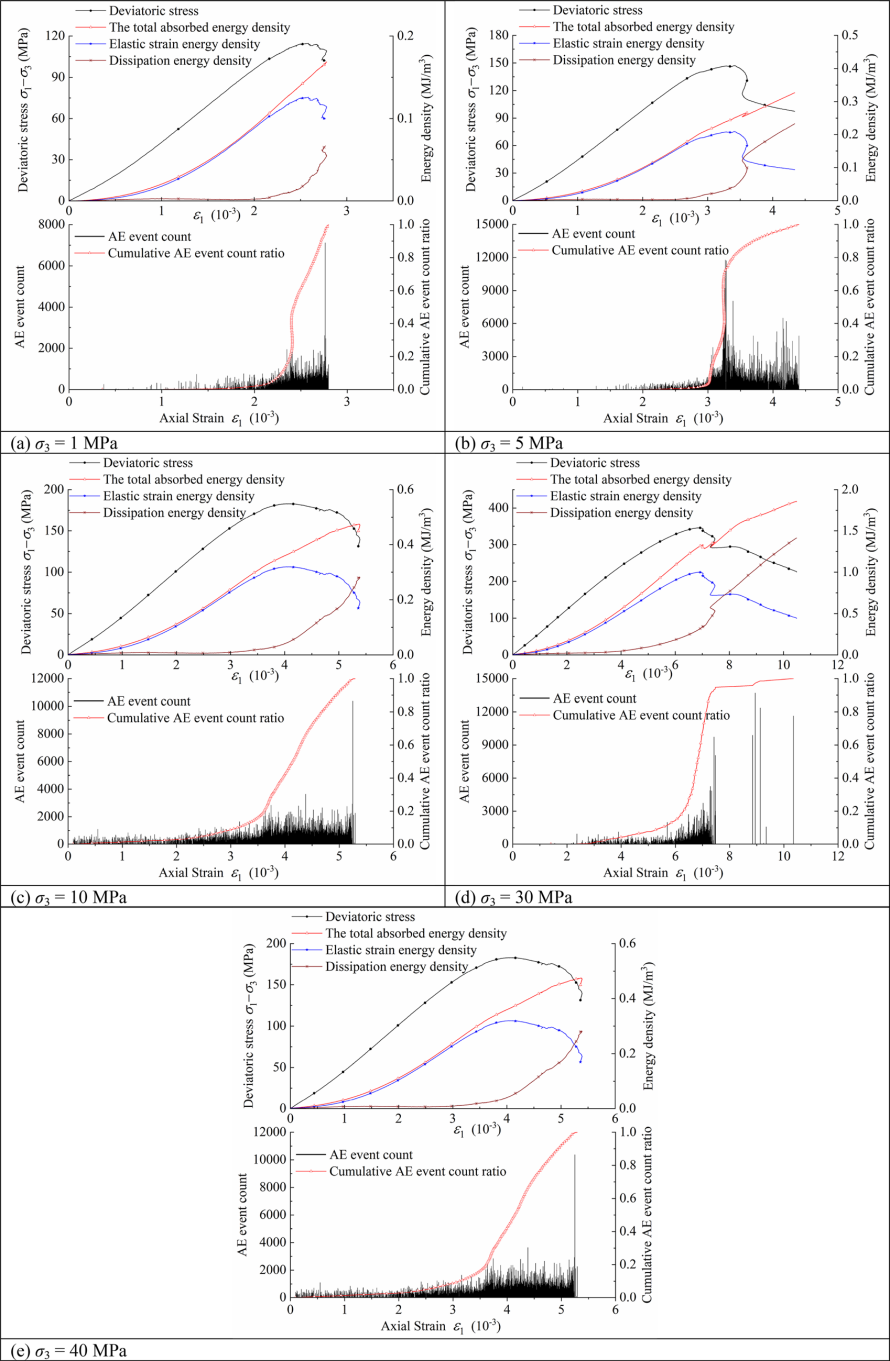
The relationship between the deviatoric stress, energy density, AE event count, cumulative AE event count ratio and axial strain of Shuangjiangkou granite samples.

Figure 6 depicts the relationship between the deviatoric stress, energy density, AE event count, cumulative AE event count ratio and axial strain of Shuangjiangkou granite samples. Both the dissipation energy density and input energy density of Shuangjiangkou granite specimens increase as axial strain increases under various confining pressures. This indicates that the test system always inputs energy to the granite sample during the whole test process, and the energy is gradually dissipated due to plastic deformation (crystal dislocation), damage evolution (nucleation and movement of microcracks), friction of the fracture surface and dilatancy caused by the roughness of the fracture surface. When the elastic strain energy density curves of five groups of granite samples are compared to the stress–strain curves under the same confining pressure, it is discovered that the elastic strain energy density variation trend is very similar to that of deviatoric stress. The elastic strain energy density rises first and then falls, reaching its maximum value at peak stress with axial strain. This demonstrates that the prepeak period is mostly concerned with the storage of elastic strain energy, whereas the post-peak stage is primarily concerned with its release. At the elastic deformation stage, AE event rarely occurs. At the stage of steady crack development, AE event count increases. At the unstable crack development stage, AE event count increases sharply. When the stress reaches the peak, AE event intensifies. At the post-peak stage, AE event count reaches its peak. The variation trend of the cumulative AE event count ratio with axial strain is basically consistent with that of dissipated energy density, which indicates that the total energy absorbed by rock causes the AE event in the process of energy dissipation.

### Energy dissipation ratio

The parameter energy dissipation ratio *χ* is introduced here, which can be calculated by Eq. (11).

11 $$\chi = \frac{{U_{d} }}{U}.$$

**Figure 7:**
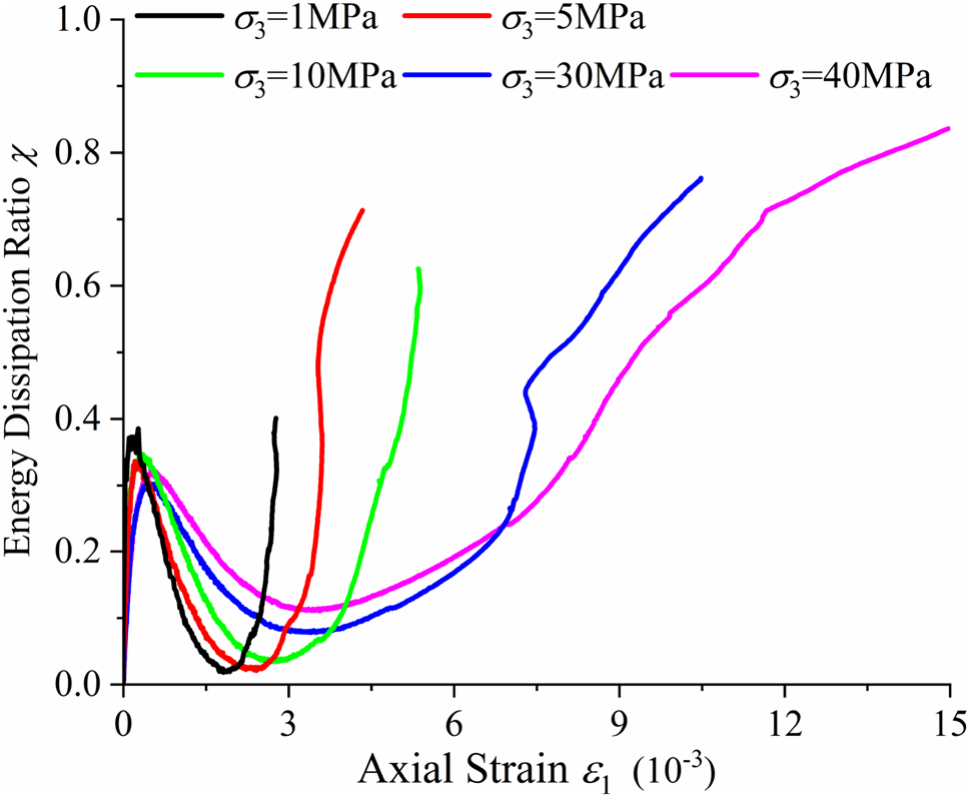
The relationship between axial strain and energy dissipation ratio under different confining pressures.

Figure 7 shows the variation trend of the energy dissipation ratio *χ* with axial strain under various confining pressures. The energy dissipation ratio has an obvious regular variation with axial strain, which first increases, then decreases and increases again. According to the five groups of curves (as shown in Fig. 7), curve fitting can be carried out for the five stages of energy dissipation ratio variation under different confining pressures; thus, the theoretical equation between the energy dissipation ratio and axial strain is obtained, as shown in Eq. (12) when the deviatoric stress (*σ*_1_–*σ*_3_) is less than the crack initiation stress *σ*_*ci*_ and in Eq. (13) when (*σ*_1_–*σ*_3_) is equal or greater than *σ*_*ci*_. In order to have a simple functional relationship between energy dissipation ratio and axial strain, as well as between energy dissipation ratio and confining pressure, the coefficients are fitted with the confining pressure, as shown in Eqs. (14) and (15).

12 $$\chi = r_{1} \times (1 - \exp ( - r_{2} \times \varepsilon_{1} )) + r_{3} \times \varepsilon_{1} ,\sigma_{1} - \sigma_{3} < \sigma_{ci} ,$$

13 $$\chi = s_{1} \times \exp (s_{2} \times \varepsilon_{1} ) + s_{3} \times \varepsilon_{1} ,\sigma_{1} - \sigma_{3} \ge \sigma_{ci} ,$$

where *r*_*i*_ and *s*_*i*_ are the relevant parameters of the theoretical equation, which can be calculated by Eqs. (14) and (15), respectively.

14 $$r_{i} = a_{i} \times (\sigma_{3} )^{2} + b_{i} \times \sigma_{3} + c_{i} ,$$

15 $$s_{i} = d_{i} \times (\sigma_{3} )^{2} + e_{i} \times \sigma_{3} + f_{i} ,$$

where *a*_*i*_, *b*_*i*_, *c*_*i*_, *d*_*i*_, *e*_*i*_ and *f*_*i*_ are the relevant parameters of the theoretical equation, as shown in Table 2.

**Table 2:** The parameters of the theoretical equations.

*σ*_1_–*σ*_3_	*r*_1_, *s*_1_			*r*_2_, *s*_2_			*r*_3_, *s*_3_		
< *σ*_*ci*_	*a*_1_	*b*_1_	*c*_1_	*a*_2_	*b*_2_	*c*_2_	*a*_3_	*b*_3_	*c*_3_
	0.00007	− 0.0040	0.4353	0.0091	− 0.6450	17.1070	− 0.0002	0.0106	− 0.2877
≥ *σ*_*ci*_	*d*_1_	*e*_1_	*f*_1_	*d*_2_	*e*_2_	*f*_2_	*d*_3_	*e*_3_	*f*_3_
	0.0001	− 0.0143	0.5557	0.0003	− 0.0209	0.5468	− 0.0007	0.0444	− 0.8054

### Stress thresholds and deformation characteristics

**Figure 8:**
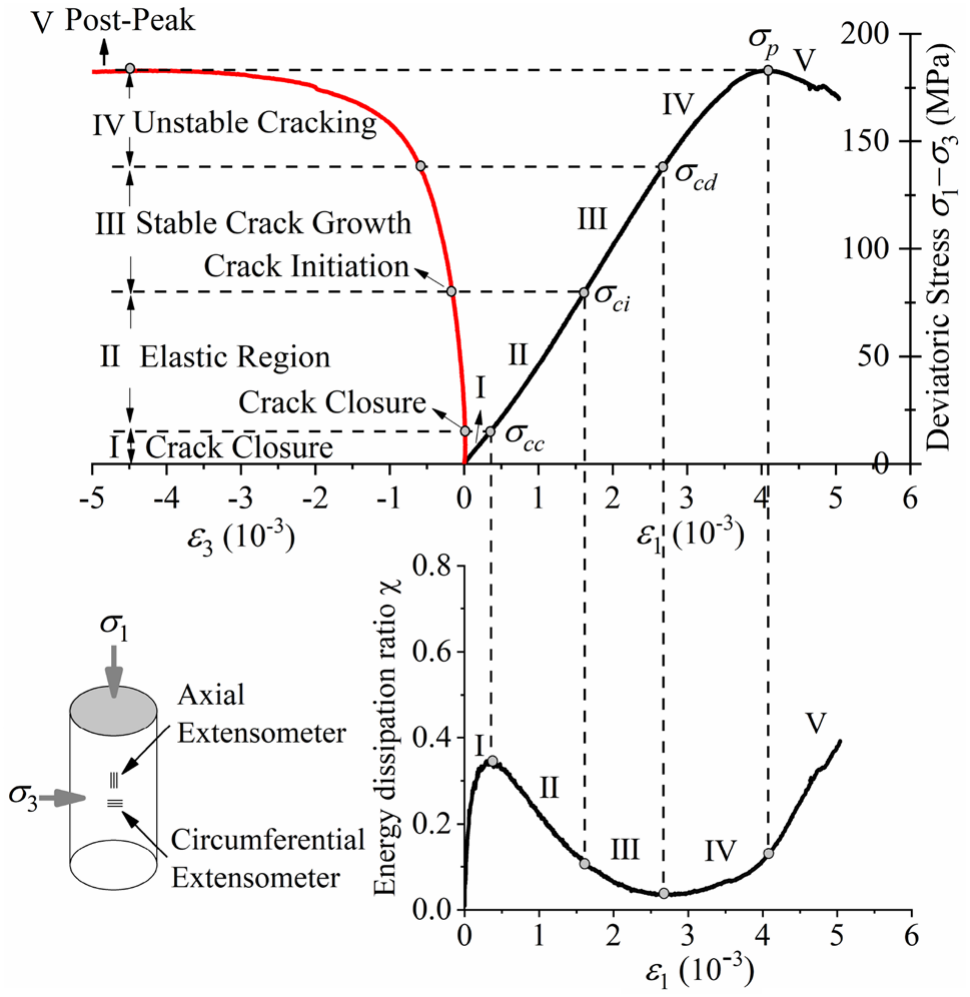
Rock failure stages based on energy dissipation rate and stress thresholds under conventional triaxial compression test (*σ*_3_ = 10 MPa).

**Figure 9:**
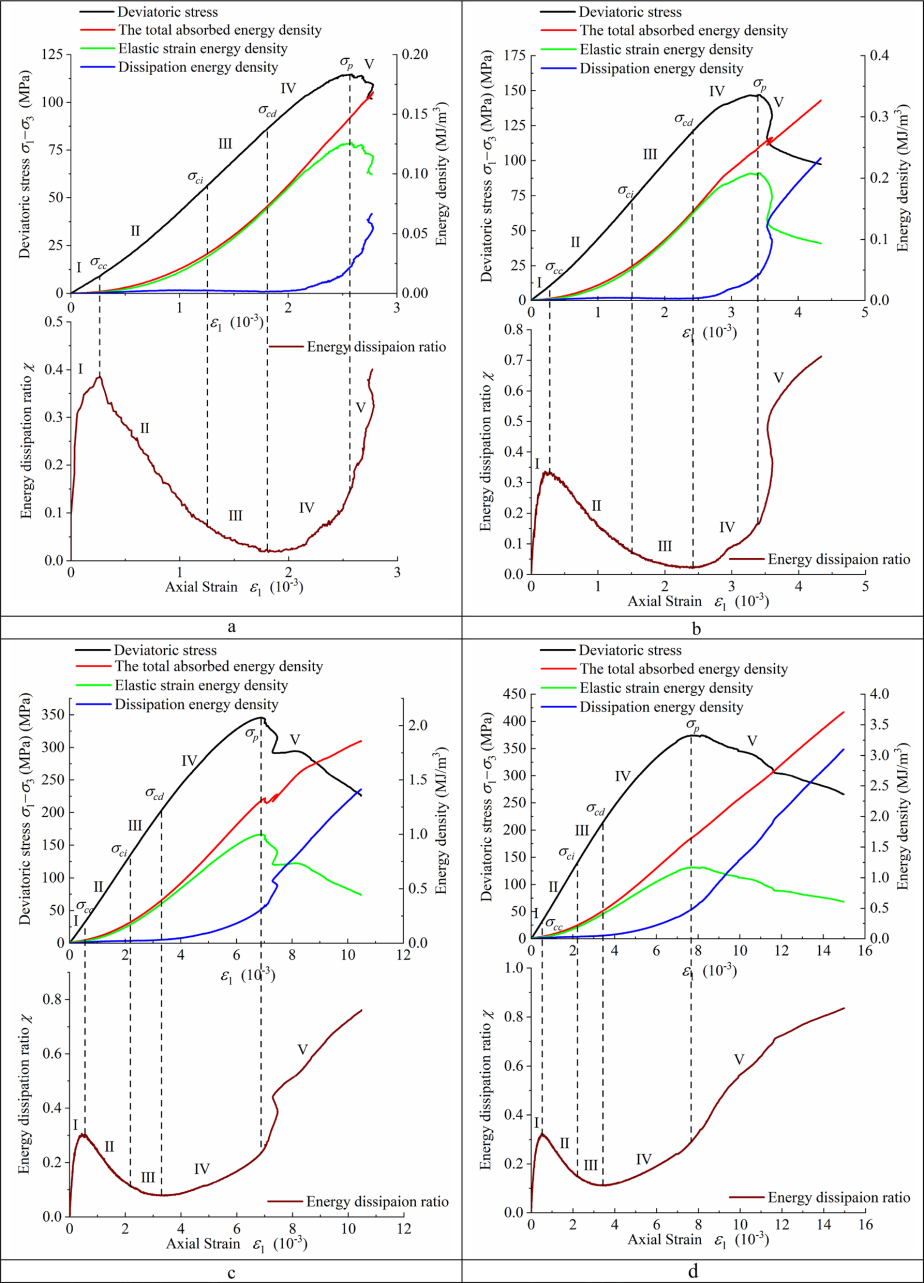
(**a**) Stages of rock failure (*σ*_3_ = 1 MPa). (**b**) Stages of rock failure (*σ*_3_ = 5 MPa). (**c**) Stages of rock failure (*σ*_3_ = 30 MPa). (**d**) Stages of rock failure (*σ*_3_ = 40 MPa).

A large number of studies^11–13,50,51^ show that a rock’s stress–strain curve can be separated into five stages by using four threshold values (i.e., *σ*_*cc*_, *σ*_*ci*_, *σ*_*cd*_, and *σ*_*p*_), as shown in Figs. 8 and 9. The ultimate stress (*σ*_*p*_) is the peak point of the stress–strain curve, and the other three threshold values can be determined in many ways. Peng et al.^51^ proposed a method to determine the crack closure stress (*σ*_*cc*_), which is easily programmed to make the determination more objective. Cai et al.^50^ proposed a method to determine the damage stress (*σ*_*cd*_) that has been proven to be relevant to jointed rock masses.

A method called the lateral strain response (LSR) method used in this paper to determine the crack initiation stress (*σ*_*ci*_) was introduced by Nicksiar and Martin^14^, which depends solely on the LSR and eliminates the need for user judgement.

A new method is proposed to determine *σ*_*cc*_ and *σ*_*cd*_ by using the relationship between the energy dissipation ratio and axial strain in this paper, as shown in Figs. 8 and 9. The stress at the curve’s crest was determined to be *σ*_*cc*_, while the stress at the curve’s trough was determined to be *σ*_*cd*_. Table 3 displays the stress thresholds of Shuangjiangkou granite samples subjected to various confining pressures.

**Table 3:** Stress thresholds of granite samples.

*σ*_3_ (MPa)	*σ*_*cc*_ (MPa)	*σ*_*ci*_ (MPa)	*σ*_*cd*_ (MPa)	*σ*_*p*_ (MPa)
1	9.10	56.75	86.66	114.76
5	10.35	71.65	120.67	147.12
10	15.19	79.80	138.69	183.04
30	27.07	134.02	202.05	345.56
40	32.71	144.92	213.82	374.17

As shown in Figs. 8 and 9, the deviatoric stress–strain curve of Shuangjiangkou granite obtained by conventional triaxial compression tests can be divided into five stages by four stress thresholds as well as the curve of the energy dissipation ratio and axial strain.

1. Region I is the compaction stage of the rock’s primary microcrack. The stress threshold corresponding to the end point is *σ*_*cc*_. The development process of rock deformation and failure is closely related to the changes in microcracks and microvoids that exist in the primary rocks. Under the condition of increasing stress, the primary microcracks and microvoids close under pressure at first, and the deviatoric stress–strain curve appears as an upper concave section. Most of the energy absorbed at the initial loading stage is stored in the rock, and a small part is dissipated due to rock compaction. However, the energy dissipation ratio increases rapidly, leading to an upwards trend of the curve.
2. Region II is the stage of linear elastic deformation. When the stress exceeds *σ*_*cc*_, the rock deformation enters the linear elastic stage. Although the axial strains and lateral strains also increase, microcracks and microvoids in the rock do not develop. Before the stress reaches *σ*_*ci*_, rock internal crystal dislocations are rare, and new microcrack nucleation and movement are also rare. Furthermore, no macroscopic surface fracture forms, so little energy is dissipated, and the majority of the energy absorbed is transformed into elastic strain energy. There is an obvious upper concave section in the elastic strain energy density curve. The energy dissipation ratio decreases, leading to a downwards trend of the curve.
3. Region III marks stable crack growth from the beginning of *σ*_*ci*_ to the end of *σ*_*cd*_, which corresponds to long-term strength^15,52^. When the stress exceeds *σ*_*ci*_, microcracks and microvoids newly generated in the rock begin to develop, some microcracks connect to each other; the number of microcracks increases; some microcracks begin to merge, cross, and then gradually form irregular longitudinal macroscopic cracks; and the increasing rate of axial strain and lateral strain increases. The crystal dislocation and the nucleation and movement of microcracks in the rock develop stably. Crystal dislocation is the basis of plastic deformation whose macroscopic expression is irreversible deformation (Fig. 4). As the lattice array of microscopic structures within the rock slips, a row of side dislocations expands from the centre of the slip band. If the motion of these linear defects is intercepted by a granular boundary, then the number of dislocations will increase near the barrier, and the dislocation accumulation near the particles increases the local stress field so that the dislocations are forced to merge and form isolation zones. When a wide enough isolation zone forms, the adjacent planes are separated, and a microfracture nucleus forms. Once a microfracture occurs, the local stress field will change automatically. The increased stress causes further dislocation slip and new dislocation accumulation at the tips of microcracks. In other parts of the microcrack, stress will be released, allowing more dislocations to enter and widen the microcrack. Despite the fact that the energy dissipation ratio continues to fall, energy dissipation begins to rise, while elastic strain energy growth slows. Only a small portion of the absorbed energy has been dissipated, and the majority of it is still transforms into elastic strain energy.
4. Region IV is the stage of unstable crack growth. When the stress exceeds *σ*_*cd*_, the crystal dislocation emission and the nucleation and movement of microcracks in the rock begins to develop rapidly. Additionally, there are a greater number of microcracks, and the microcracks begin to merge, cross, gradually form irregular macroscopic crack. A macroscopic fracture surface begins to appear, and the rock enters the stage of rapid crack development, while work hardening can be observed. The speed of energy dissipation increases rapidly, and the curve of the energy dissipation ratio shows a clear upwards trend, while the growth rate of the elastic strain energy clearly slows.
5. Region V is the post-peak failure stage. As the stress exceeds the ultimate stress (*σ*_*p*_), the rock failed rapidly. Moreover, a number of relatively regular macroscopic cracks are formed inside the rock; these macroscopic cracks are connected to form one or more macroscopic fracture surfaces with a dip angle of less than 45° to the principal stress direction (Fig. 4). This behaviour is a typical failure under compressive stress and is generally described as compression-shear failure characterized by shear deformation along the fracture plane. Heat is generated by the intense friction of the macroscopic fracture surface; and the roughness of macroscopic fracture surfaces leads to obvious dilatancy. All of these factors cause the energy dissipation ratio to rapidly increase, and the curve shows a sharp upwards trend. After reaching the peak, the accumulated elastic strain energy is quickly released and then dissipated by converting it into various forms of energy, aggravating rock failure.

## Nonlinear statistical damage constitutive model

### Damage variable

Statistical approaches can be used to represent the properties of rock on the mesoscale. Assume that a rock is made up of many meso-elements that are large enough to contain many microdefects. In comparison to the whole structure of the rock, the size of the meso-element is deemed to be appropriately minute. Therefore, the significant impact of a single microdefect can be ignored. This indicates that each meso-element can be considered a particle in rock in the context of continuum mechanics theory. It is then possible to describe rock damage and failure behaviour in terms of these meso-elements. Rock, which has obvious heterogeneity, is composed of one or several kinds of minerals. Native rock is filled with a random distribution of various microdefects, which are caused by nucleation and random movement of microcracks under loading, and the characteristics of the mechanical properties of microdefects show a random distribution, so the damage produced by the mesoscopic elements is also randomly distributed in the rock material. This indicates that rock’s macroscopic characteristics are determined by their meso-element statistical properties.

Equation (16) defines the damage variable that describes the granite damage characteristics.

16 $$D = \frac{{N_{D} }}{N},$$

where *N*_*D*_ is the number of damaged meso-elements and *N* is the total number of meso-elements.

Considering that the damage of rock material in the loading process is a continuous process, the following simplified assumptions are made before modelling the rock: (1) rock can be regarded as an isotropic, homogeneous, continuous and brittle material in which there are microcracks and microvoids at the macroscale; (2) the meso-elements of rock obey Hooke’s law before failure; that is, the meso-elements have linear elastic properties, and the elastic damage constitutive equation applies to each meso-element; and (3) the damage of rock is progressive, which is the gradual accumulation of the damage of the meso-elements.

As described in “Energy evolution and deformation characteristics”, the energy dissipation mechanism of rock is very complex. Factors such as crystal dislocation, nucleation and movement of microcracks, frictional heat generation of macroscopic fracture surfaces, and dilatancy caused by roughness of macroscopic fracture surfaces all lead to the gradual dissipation of energy absorbed by rock from the outside, and energy dissipation occurs at the same time as rock damage evolution. The process of rock damage evolution causes energy dissipation. The study of rock damage evolution features from the standpoint of energy dissipation can, in essence, reflect the deformation and failure process of rock. The energy dissipation induced by damage is intrinsically random due to the random distribution of damage of meso-elements in rock material.

According to the previous analysis, the energy dissipation ratio has the characteristics of a random distribution. Suppose that the energy dissipation ratio (χ) follows a Weibull distribution^36^; therefore, the probability density function of the energy dissipation ratio can be expressed by Eq. (17).

17 $$P\left( \chi \right) = \frac{k}{m}\left( {\frac{\chi }{m}} \right)^{k - 1} \exp \left[ { - \left( {\frac{\chi }{m}} \right)^{k} } \right],$$

where *P*(χ), *m*, and *k* are functions of probability density, scale parameter and shape parameter, respectively.

The degree of energy dissipation of rock material when subjected to external force also indicates the degree of damage. Equation (18) can be used to express the total number of damaged meso-elements as the dissipated energy density grows.

18 $$N_{D} = N\int {P\left[ \chi \right]} {\text{d}}\left( \chi \right).$$

Substituting Eq. (17) into Eq. (18) yields Eq. (19).

19 $$N_{D} = N\left\{ {1 - \exp \left[ { - \left( {\frac{\chi }{m}} \right)^{k} } \right]} \right\}.$$

Substituting Eq. (19) into Eq. (16) yields Eq. (20).

20 $$D = 1 - \exp \left[ { - \left( {\frac{\chi }{m}} \right)^{k} } \right].$$

The damage evolution equation for meso-elements in granite is Eq. (20).

### Nonlinear statistical damage constitutive model

Strain and nominal stress can be directly measured experimentally in conventional triaxial compression tests of rock. According to the Lemaitre strain equivalence principle^31^, the damaged material’s constitutive equation is the same as that of the original material, with the exception that the nominal stress is substituted with the effective stress. Equation (21) shows the relationship between the effective and nominal stresses.

21 $$\sigma_{i} = \tilde{\sigma }_{i} \left( {1 - D} \right),\quad i = 1 \sim 3,$$

where $\sigma_{i}$ and $\tilde{\sigma }_{i}$ are the nominal and effective stresses, respectively.

The theory of deformation of porous materials containing a viscous fluid proposed by Biot^53^ constituted an extension of Terzaghi’s original one dimensional theory^54^ and Hooke’s law, which is shown in Eq. (22).

22 $$\varepsilon_{i} = \frac{{\tilde{\sigma }_{i} }}{E} - \frac{\nu }{E}\left( {\tilde{\sigma }_{j} + \tilde{\sigma }_{k} } \right) - \frac{p}{3H},\quad i,j,k = 1 \sim 3,$$

where *p* is pore pressure, 1/*H* is an additional physical constant which is a measure of the compressibility of the soild for a change in water pressure.

Substituting Eq. (21) into Eq. (22) yields Eq. (23).

23 $$\varepsilon_{i} = \frac{{\sigma_{i} - \nu \left( {\sigma_{j} + \sigma_{k} } \right)}}{{E\left( {1 - D} \right)}} - \frac{p}{3H}.$$

Then, substituting Eq. (20) into Eq. (23) yields Eq. (24).

24 $$\sigma_{i} = \nu \left( {\sigma_{j} + \sigma_{k} } \right) + E\left( {\varepsilon_{i} + \frac{p}{3H}} \right)\exp \left[ { - \left( {\frac{\chi }{m}} \right)^{k} } \right].$$

Equation (24) is the nonlinear statistical damage constitutive model of Shuangjiangkou granite that takes into account the influence of pore pressure. When the pore pressure is not considered, Eq. (24) under conventional triaxial compression test conditions degenerates into Eq. (25).

25 $$\sigma_{1} = 2\nu \sigma_{3} + E\varepsilon_{1} \exp \left[ { - \left( {\frac{\chi }{m}} \right)^{k} } \right].$$

### Weibull distribution parameters

To establish the nonlinear statistical damage constitutive equation of Shuangjiangkou granite, it is necessary to determine *m* and *k*. Eq. (26) is obtained by two logarithmic operations on Eq. (24).

26 $$\ln \left[ { - \ln \left( {\frac{{\sigma_{i} - \nu \left( {\sigma_{j} + \sigma_{k} } \right)}}{{E\left( {\varepsilon_{i} + \frac{p}{3H}} \right)}}} \right)} \right] = k\ln \left( \chi \right) - k\ln m.$$

When the pore pressure is not considered, Eq. (26) under conventional triaxial compression test conditions degenerates into Eq. (27).

27 $$\ln \left[ { - \ln \left( {\frac{{\sigma_{1} - 2\nu \sigma_{3} }}{{E\varepsilon_{1} }}} \right)} \right] = k\ln \left( \chi \right) - k\ln m.$$

Equation (28) is the simplest linear equation, which is simplified from Eq. (26).

28 $$Y = kX + b.$$

The parameters of Eq. (28) are represented in Eq. (29).

29 $$\left\{ \begin{gathered} X = \ln \left( \chi \right) \hfill \\ Y = \ln \left[ { - \ln \left( {\frac{{\sigma_{i} - \nu \left( {\sigma_{j} + \sigma_{k} } \right)}}{{E\left( {\varepsilon_{i} + \frac{p}{3H}} \right)}}} \right)} \right] \hfill \\ b = - k\ln m. \hfill \\ \end{gathered} \right.$$

When the pore pressure is not considered, *Y* can be calculated by Eq. (30).

30 $$Y = \ln \left[ { - \ln \left( {\frac{{\sigma_{1} - 2\nu \sigma_{3} }}{{E\varepsilon_{1} }}} \right)} \right].$$

The parameters *k* and *b* can be calculated from a series of triaxial test data. The parameters related to the experiments without considering the influence of pore pressure are shown in Table 4.

**Table 4:** Weibull distribution parameters.

Confining pressure (MPa)	*k*	*m*
1	1.11	0.81
5	0.70	0.87
10	0.71	1.33
30	0.98	1.11
40	1.49	0.68

### Verification of the constitutive model

**Figure 10:**
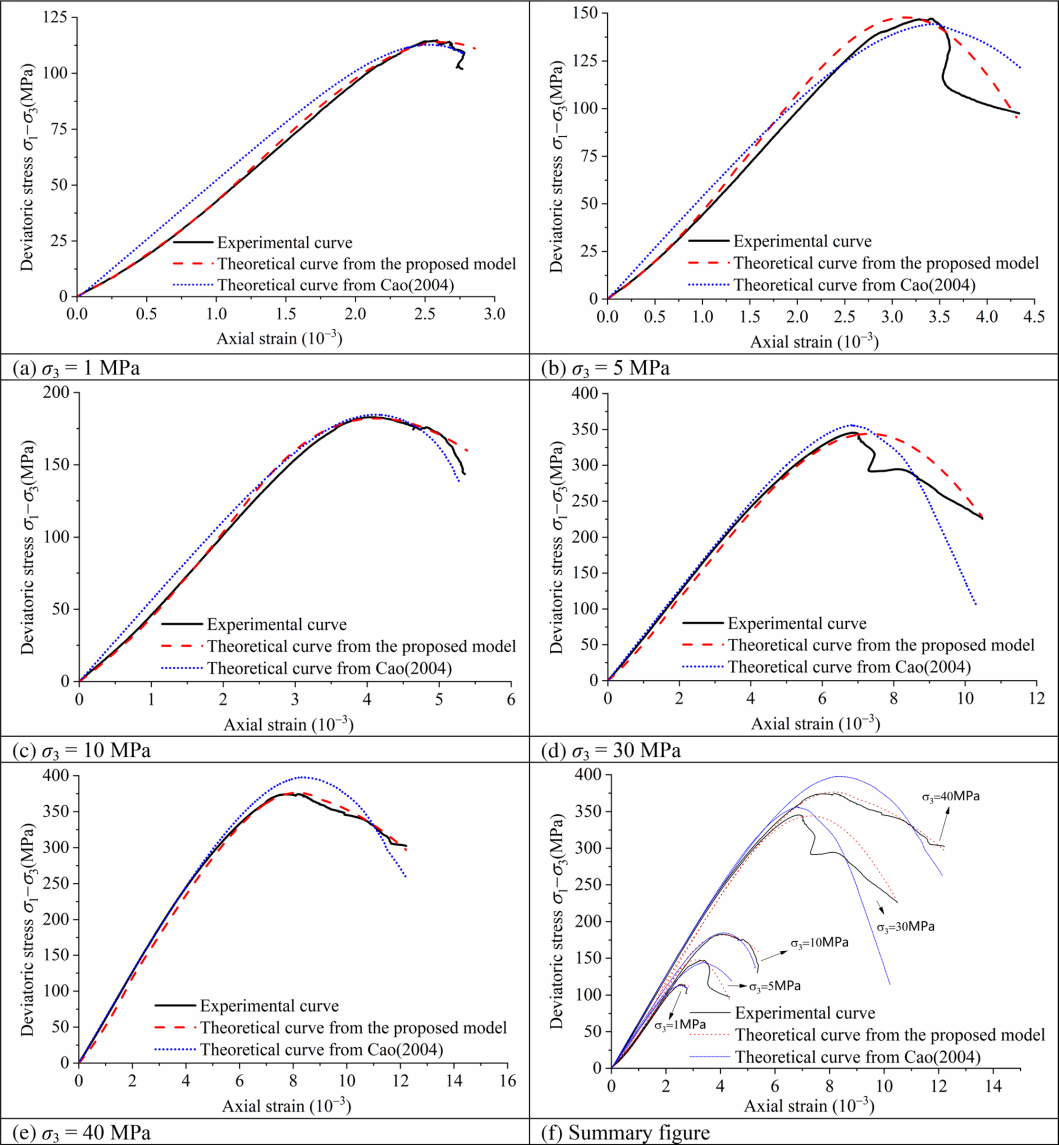
The relationship curve of the deviatoric stress-axial strain and theoretical deviatoric stress-axial strain of Shuangjiangkou granite under various confining pressures.

The comparison between the theoretical curves of the model proposed and the model from Cao^55^ is shown in Fig. 10, in which also shown the experimental curves under various confining pressures. In the case of low confining pressure (as shown in Fig. 10a–c), the fitting effect of the model proposed in this paper is better than that of literature^55^ at the pre-peak stage. In the case of high confining pressure (as shown in Fig. 10d,e), the fitting effect of the model proposed in literature^55^ is slightly better at the elastic stage. However, the model proposed in this paper has better overall fitting effect. Due to the existence of confining pressure, the rock does not lose its ability to bear load immediately after reaching the peak strength, but there is a process of stress and strain readjustment, which leads to the unstable snap-back phenomenon of the test curve. Both models failed to simulate the phenomenon well, so more research is needed to solve the problem.

The variation trend of the curves based on the theoretical model proposed is similar to that obtained from the experimental data, indicating that the theoretical constitutive model reflects the genuine constitutive relationship of granite, according to the analysis of the five sets of curves. The principles of elasticity, continuum mechanics, strain equivalence, and statistical analysis all serve as the theoretical foundation for the theoretical constitutive model. There are some theoretical assumptions and simplified processing when processing five groups of fixed confining pressure conventional triaxial test data through a series of mathematical analyses.

## Conclusion

In this paper, MTS815 rock test equipment was used to carry out five groups of conventional triaxial tests on Shuangjiangkou granite, and the following conclusions were drawn after study:

1. Both the total input and dissipation energy density of Shuangjiangkou granite samples increase as the axial strain increases, whereas the trend of the elastic strain energy density is highly similar to that of the deviatoric stress. The variation of AE event count can predict the failure process of Shuangjiangkou granite.
2. The fitting formula of the energy dissipation ratio is given based on the curves of the energy dissipation ratio-strain under various confining pressures, and a new approach for calculating the crack closure stress and damage stress based on the energy dissipation ratio is provided.
3. A new nonlinear statistical damage constitutive model of granite is developed based on the energy dissipation ratio. The proposed model based on energy dissipation rate can provide reference for relevant scientific research.

The deformation of brittle rock is inherently time dependent^56–58^, The model proposed didn’t consider the effects of temperature and time, which requires more researches. The strength of granite given in this paper is short-term strength, however, the authors’ another paper^59^ studied the creep properties of granite collected from the same site. Therefore, the proposed model based on energy dissipation rate can provide reference for relevant scientific research, and when applying this model to guide engineering design, the influence of pore pressure must be considered, in addition, the effects of temperature and time should also be taken into account.

## Data Availability

All data generated or analysed during this study are included in this published article.
